# Management of chronic open-angle glaucoma

**Published:** 2022-01-31

**Authors:** Heiko Philippin

**Affiliations:** 1 Clinical Research Fellow: International Centre for Eye Health, London School of Hygiene & Tropical Medicine, UK. Global Advisor for Inclusive Eye Health/Research & Training: CBM, Bensheim, Germany and Glaucoma Specialist: Eye Center, Medical Center, University of Freiburg, Germany.

**The rate of progression is the deciding factor in when and how to treat primary open-angle glaucoma. Treatment is complex, so it is important to consider patient and health care factors while keeping in mind the overall aim: preserving the patient’s quality of life and livelihood.**

**Figure:**
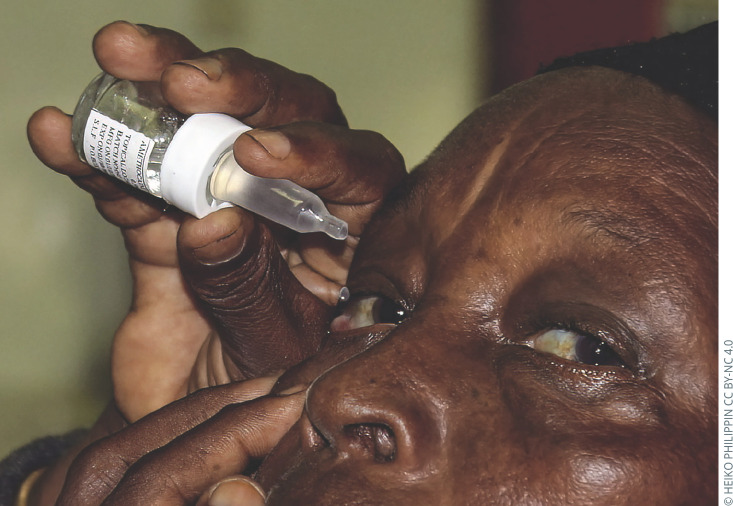
It is important that glaucoma patients learn how to instil any prescribed eyedrops correctly. **TANZANIA**

The purpose of glaucoma care is to preserve the quality of life and livelihood of a person with glaucoma, which includes maintaining their visual function while minimising the side-effects and complications of treatment. In order to deliver such patient-centred care, a glaucoma care system which can provide long-term, affordable, sustainable, and equitable care needs to be in place.

## The objective of glaucoma treatment

A person with open-angle glaucoma is at risk of irreversible blindness. The objective of treatment is to minimise this risk, usually by lowering the intraocular pressure (IOP) so that an individual upper threshold IOP (also known as their target IOP) is not exceeded. However, we must weigh the expected long-term benefit of preserving vision against side-effects, complications, and the long-term cost of treatment – all of which can affect quality of life and the person’s livelihood.

**Key points**

- The aim of patient-centred glaucoma care is to preserve and promote quality of life and livelihood
- The objective of treatment is to minimise the risk of irreversible vision loss
- The individual therapy plan is determined by the rate of progression of glaucoma
- Available treatments should be tailored to the person with glaucoma
- A single measurement of a high intraocular pressure (IOP) alone should not usually trigger a change of the plan
- Refer patients for low vision care, rehabilitation, and counselling as needed.

## Choosing a therapy plan

An individual therapy plan is based on a detailed history, visual acuity, and examination of general and glaucoma-related structural and functional details and any changes in these (visual field, disc damage likelihood scale, etc.).

The key result from the history and examination is the rate of progression of the glaucomatous damage. This has to be determined regularly, for each eye separately, and can be divided broadly into three groups:

**Group 1.** Probably no progression, or only a low rate of progression.

**Group 2.** Insufficient information to determine the rate of progression.

**Group 3.** A high rate of progression of vision loss which will probably lead to vision impairment during the patient’s’ lifetime and might have an impact on her or his quality of life and daily activities.

If the rate of progression is low, monitoring can continue, either by only observing the eye or continuing with the same treatment (Group 1). If this was the patient’s first assessment or if there is not enough information from previous examinations available, the risk for progression can be estimated (Group 2). An increased risk of glaucoma progression to visual loss is associated with advanced disease on presentation, high intraocular pressure, older age, certain ethnic groups, disc haemorrhages, and thin central corneal thickness, among others.^1^^,^^2^

If there is an estimated high risk of progression (Group 2), or if there is actual evidence of a high rate of progression (Group 3), an escalation of treatment is indicated. However, it is important to review the current treatment before escalating the therapy; e.g., first checking whether the patient was able to purchase the prescribed eye drops and whether they have actually been taking the treatment.

## Lowering IOP

Lowering IOP prevents or delays the onset or progression of glaucoma. However, there is no specific IOP threshold, formula, or percentage reduction which applies to all patients. Instead, it is recommended to set and subsequently adapt an **individual target IOP**. This can be defined as the IOP that slows down the rate of progression of the glaucomatous damage enough to maintain the patient’s quality of life and livelihood during their lifetime.^1^^,^^3^

This definition contains three elements which need to be considered:

- Intraocular pressure
- Rate of progression of the glaucomatous damage
- Quality of life and livelihood.

Analysis of the advanced glaucoma intervention study (AGIS) showed that participants with IOP <18 mmHg at 100% of visits showed no visual field progression.^4^^,^^5^ However, high-quality prospective data comparing different target IOP levels are not currently available; as such, the trade-off between risks and benefits associated with different thresholds is unclear.^1^ Target pressure should therefore be individualised and may need adjustment over time.^1^

A single measurement of a high intraocular pressure alone should not trigger a change of management and needs to be put into the context of the other examination results and the history, including self-reported adherence. IOP may also fluctuate within hours or days so that several measurements might provide a better picture of the general level of IOP in an eye. Sometimes a repeat examination on the same day or a repeat follow-up visit within a few weeks might be helpful to decide on the next step, e.g., an escalation of treatment. This also depends on the level of urgency, which can be high for eyes with severe visual field loss and a high rate of progression.

There are several treatment options available to reduce IOP. They can be divided in three groups: medical treatment (usually eye drops but may include oral or intravenous medication, e.g., acetazolamide), laser and surgery. Current cost-effective examples are timolol eye drops, selective laser trabeculoplasty, and trabeculectomy. Other eye drops are only available at considerably higher cost and may not be affordable for some patients in an LMIC context.^6^

Some examples are given below, but these will vary depending on the local or regional glaucoma care system.

## Medical treatment

Medication (a conservative treatment) can reduce IOP by decreasing aqueous production (Table 1a) or enhancing aqueous outflow (Table 1b). Osmotic agents are not mentioned as they are not for long-term use.

**Table 1a:** Efficacy and side effects of glaucoma medication to decrease aqueous production

Drug	Efficacy	Side effects (selection)
**β-Blockers** (e.g., timolol)	+++	Bronchospasm, bradycardia, depression
**Carbonic anhydrase inhibitors (systemic)** (e.g., acetazolamide)	++++	Metallic taste, electrolyte imbalance
**Carbonic anhydrase inhibitors (topical)** (e.g., dorzolamide)	++	Stinging, burning, headache
**α2-adrenergic agonists** (e.g., brimonidine)	++(+)	Toxic reaction of external eye, dry mouth. Contraindicated in children

**Table 1b:** Efficacy and side effects of glaucoma medication to enhance aqueous outflow

Drug	Efficacy	Side effects (selection)
**Prostaglandin analogues** (e.g. latanoprost)	+++(+)	Eyelash growth, periorbital fat atrophy, increased iris pigmentation
**Rho-kinase inhibitors** (e.g. netarsudil)	++(+)	Conjunctival hyperaemia, headache
**Cholinergic agonists** (e.g., pilocarpine)	++(+)	Headache, dim vision

## Laser treatment

Laser treatment can decrease aqueous production by partial destruction of the ciliary body epithelium, which produces aqueous (Table 2a) or by increasing aqueous outflow through the trabecular meshwork (Table 2b).

**Table 2a:** Laser treatment to decrease aqueous production

Laser	Comments
Transscleral cyclophotocoagulation (TSCPC)	Typically, diode laser (810 nm) is used. Risk of irreversible hypotony. Therefore, fractional treatment is common.^7^
Endoscopic cyclophotocoagulation	Similar to TSCPC, with a better complications profile, but more invasive.
Micropulse transscleral cyclophotocoagulation (MP-TSCPC)	Diode laser (810 nm) with short bursts instead of continuous delivery of laser energy to reduce destruction of adjacent non-ciliary tissue. Might also enhance uveoscleral outflow.^8^

**Table 2b:** Laser treatment to enhance aqueous outflow

Laser	Comments
Argon laser trabeculoplasty (ALT)	Initial treatment with argon laser trabeculoplasty was at least as efficacious as initial treatment with topical medication (GLT). Risk of scarring of the trabecular meshwork and peripheral anterior synechiae formation.
Selective laser trabeculoplasty (SLT)	532 nm frequency-doubled Q-switched Nd:YAG laser. Similar efficacy as ALT (LiGHT, KiGIP SLT trials) but less side effects and repeatable.^9^^,^^10^
Micropulse laser trabeculoplasty (MLT)	Using 810 nm, 532 nm or 577 nm lasers. Possibly similar efficacy as SLT

## Surgery

There are several surgical options to reduce intraocular pressure, including a selection of minimally invasive options. ‘Ab externo’ refers to a surgical approach from outside the eye, often involving a conjunctival dissection and scleral incision. ‘Ab interno’ refers to a surgical approach from inside the eye, usually through the anterior chamber, with a corneal incision.

There are three main categories of glaucoma surgery, each with a different purpose:

1. To enhance aqueous outflow into the sub-Tenon space
2. To enhance aqueous outflow through the trabecular meshwork
3. To enhance aqueous outflow through the suprachoroidal space.

### 1. Surgery to enhance aqueous outflow into the sub-Tenon space

Ab externo approach:

- **Trabeculectomy.** The gold standard, low-cost procedure to create a guarded fistula between the anterior chamber and sub-Tenon space, requires adherence to follow-up. The Moorfields safer technique (i.e., using releasable sutures), is also suitable in low-resource settings.^11^
- **Glaucoma drainage devices.** Aravind Aurolab drainage implant, Ahmed valve, Baerveldt shunts (250/350), PAUL Glaucoma Implant.
- **PreserFlo Microshunt.** An aqueous shunt between the anterior chamber and sub-Tenon’s space; drains more posteriorly.

**Evidence for selective laser trabeculoplasty**

The LiGHT trial in the UK^10^ showed that selective laser trabeculoplasty (SLT) as first-line treatment of ocular hypertension and primary open-angle glaucoma was safe, cost-effective and resulted in the same quality of life (after 3 years) compared to eye drops.

The Kilimanjaro Glaucoma Intervention Programme (KiGIP) SLT trial compared SLT and Timolol eye drops (with standardised counselling) in patients with moderate and advanced glaucoma in Tanzania.^11^ After one year, SLT treatment was successful in 60.7% of eyes, and Timolol eye drops were successful in 31.3% of eyes. In the SLT group, approximately one third of eyes required one repeat session of SLT; in the Timolol group, a similar proportion needed one repeat session of counselling. Safety, acceptance, vision-related quality of life, and preservation of visual acuity were comparable in both groups after one year. Eye care units in the region using a not-for-profit eye care service model would need to treat around 500 eyes per year with SLT to cover the cost of the procedure, charging an amount similar to one year’s supply of timolol eye drops.

Ab interno approach:

- **XEN gel stent.** A 6 mm porcine-derived gelatin tube with an inner lumen of 45 µm and outer diameter of 150 µm.

### 2. Surgery to enhance aqueous outflow through the trabecular meshwork

Ab externo approach:

- **Canaloplasty.** Dilation of Schlemm’s canal using viscoelastics and a suture.
- **Trabeculotomy.** Accessing Schlemm’s canal via a partial scleral flap. A curved probe (trabeculotome) is rotated gently into the anterior chamber to incise through the trabecular meshwork.
- **Deep sclerectomy.** Non-penetrating surgery otherwise similar to trabeculectomy.
- **Iridectomy.** Improving aqueous flow from the posterior to the anterior chamber.

Ab interno approach:

- **iStent.** A 360 µm stent with a central lumen of 80 µm implanted into the trabecular meshwork.
- **Hydrus.** A permanent, 8 mm long, slightly curved microstent to dilate Schlemm’s canal.
- **Goniotomy.** Typically used for childhood glaucoma. The trabecular meshwork is incised under direct gonioscopic visualisation using a goniotomy knife (e.g. 25-gauge needle on a syringe).
- **Kahook Dual Blade or Trabectome.** Disposable ab interno trabeculectomy devices to remove parts of the trabecular meshwork.

### 3. Surgery to enhance aqueous outflow through the suprachoroidal space

These include:

- **STARflo**, **Gold Micro Shunt**. Implants to access the suprachoroidal space (an ab externo approach).
- **iStent supra.** A 4-mm long curved stent with a lumen of 0.165 mm inserted into the suprachoroidal space (an ab interno approach).

## Focus on the patient, not just the eye

The variety of treatment options available makes it much easier to find an approach to suit the individual patient. There are many factors to consider, including those related to the individual and the health system; see the online version of this article (**bit.ly/CEHJpoag**) for more detail. It is just as important to ensure that people with glaucoma receive counselling to support their compliance with treatment and quality of life, and to refer them for low vision services and rehabilitation as needed – see the rest of this issue for more information.

**How to choose the right treatment for each patient**

The variety of treatment options available enables glaucoma care providers to find a treatment, or a combination of treatments, tailored to the person with glaucoma and adjusted to the available glaucoma care in the region.

**Figure 1:**
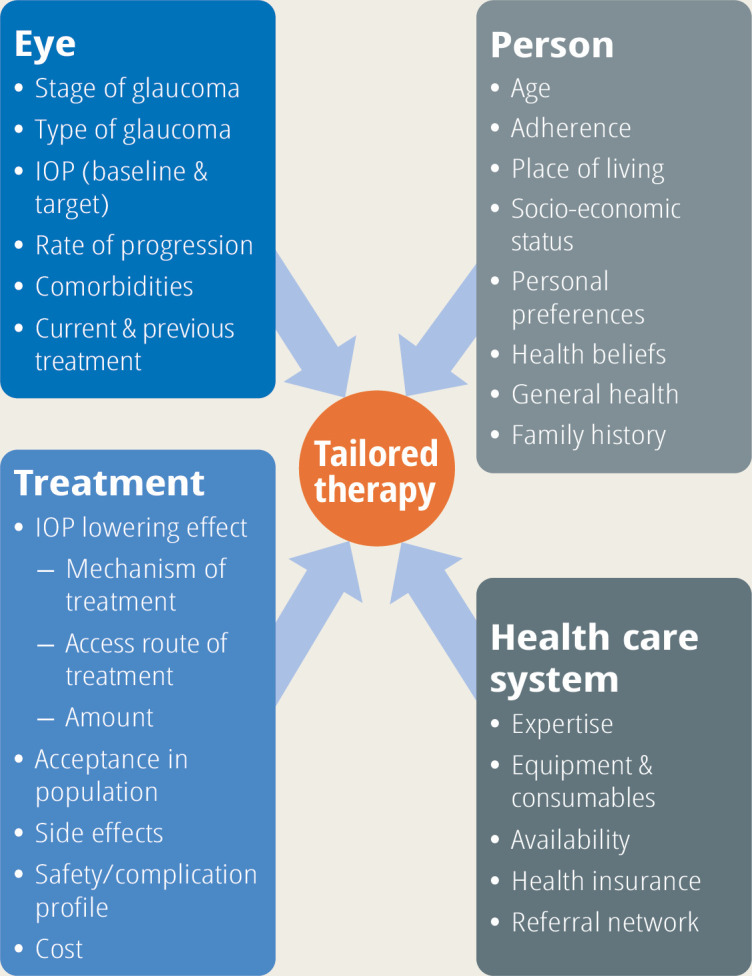
Four key groups of factors determining the individual treatment tailored to a person with glaucoma. (IOP = intraocular pressure)

When choosing the best treatment, or combination of treatments, possible for a glaucoma patient, the many factors we must consider can be usefully grouped into the following areas (see Figure 1):

1. Eye-related factors
2. Person-related factors
3. Treatment-related factors.
4. Health system-related factors

**Eye-related factors**

**Type of glaucoma.** Certain types of glaucoma are more aggressive (e.g,. exfoliation glaucoma) or might affect central vision faster and require a particularly low target IOP when the rate of progression is high, despite an intraocular pressure in the normal range.

**Stage of glaucoma.** Consider both eyes, severity of glaucoma can be asymmetric. More advanced disease is at higher risk for progression.

**IOP.** Baseline IOP and target IOP are important to determine the need for IOP reduction.

**Rate of progression.** The rate of progression is the most important factor which determines the need for treatment or escalation of treatment. If it is not yet known, there are certain risk factors which can help to estimate the rate of progression.

**Comorbidities.** Is the eye affected by other conditions, e.g., diabetic retinopathy, trauma, etc.?

**Current & previous treatments.** Which treatments are currently used? Which eye drops were unsuccessful in the past? How did the patient react to surgery before?

**Person-related factors**

**Age.** Chronological and biological age might differ, leading to different decisions. Life expectancy should be considered and depends on several factors, e.g., region, sex.

**Preferences.** Preferences of a person towards certain treatments can affect adherence.

**Health beliefs.** Certain (health) beliefs might imply specific behaviour or preferences which can be very important to the patient

**Adherence.** What has adherence to follow-up visits and topical treatment been like in the past?

**General health.** Physical ability to open eye drop bottles and administer eye drops? Side-effects or interactions with other medications or health conditions? Mobility?

**Place of living.** Determines travel distance, local availability of treatment, e.g., eye drops

**Family history.** Are other family members affected by glaucoma? Do they need to be examined?

**Socio-economic status.** Social status: need for assistance? Assistance available at home? Does the family depend on the patient, e.g., for income? Can the treatment be paid for and for how long? Does the patient have health insurance?

**Health system-related factors**

**Expertise.** Level of necessary surgical skills available? Audits or self-audits of outcomes done?

**Equipment needed.** Laser equipment? Surgical instruments, consumables? Repair and maintenance done regularly?

**Availability.** Are eye drops available at the place of living of the patient? Consumables for surgery available?

**Health insurance.** Is health insurance available and could this be recommended to the patient?

**Referral network.** Is a referral network available? Distance of other facilities? Can follow-up be delegated? Which eye drops are offered?

**Treatment-related factors**

**IOP lowering effect.** Is the treatment able to reach the target IOP or below? Duration of the effect?

**Acceptance.** How likely is it that this type of treatment will be accepted by the patient (and in the target population)?

**Side-effects.** Risk for long-term side-effects? Side-effect profile of treatment.

**Complications.** Risk of complications? E.g., bleb failure or hypotony?

**Cost.** Consider initial cost, cumulative long-term cost, follow-up cost (e.g., to treat complications).

**Main mode of IOP lowering.** Decreasing aqueous production or enhancing aqueous outflow, ab interno or ab externo approach, draining aqueous through trabecular meshwork or sub-Tenon’s?
